# Effect of celecoxib in treatment of burn-induced hypermetabolism

**DOI:** 10.1042/BSR20191607

**Published:** 2020-04-29

**Authors:** Shubo Zhuang, Jiake Chai, Lingying Liu, Huinan Yin, Yonghui Yu

**Affiliations:** 1 Department of Dermatology and Plastic Surgery, The First Hospital of Tsinghua University, Beijing, China; 2 Burn Institute, Department of Burn and Plastic Surgery, Fourth Medical Center of PLA General Hospital, Beijing, China

**Keywords:** BAT, Burn injury, Celecoxib, Hypermetabolism, UCP1

## Abstract

**Background:** Cyclooxygenase-2 (COX-2) catalyzes the rate-limiting step of prostanoid biosynthesis. Under pathologic conditions, COX-2 activity can produce reactive oxygen species and toxic prostaglandin metabolites that exacerbate injury and metabolic disturbance. The present study was performed to investigate the effect of Celecoxib (the inhibitor of COX-2) treatment on lipolysis in burn mice.

**Methods:** One hundred male BALB/c mice were randomly divided into sham group, burn group, celecoxib group, and burn with celecoxib group (25 mice in each group). Thirty percent total body surface area (TBSA) full-thickness injury was made for mice to mimic burn injuries. Volume of oxygen uptake (VO_2_), volume of carbon dioxide output (VCO_2_), respiratory exchange ratio (RER), energy expenditure (EE), COX-2 and uncoupled protein-1 (UCP-1) expression in brown adipose tissue (BAT) were measured for different groups.

**Results:** Adipose tissue (AT) activation was associated with the augmentation of mitochondria biogenesis, and UCP-1 expression in isolated iBAT mitochondria. In addition, VO_2_, VCO_2_, EE, COX-2, and UCP-1 expression were significantly higher in burn group than in burn with celecoxib group (*P*<0.05).

**Conclusion:** BAT plays important roles in burn injury-induced hypermetabolism through its morphological changes and elevating the expression of UCP-1. Celecoxib could improve lipolysis after burn injury.

## Introduction

Adipocytes can be broadly divided into white and brown fat cells. White fat cells are specialized to store chemical energy, while brown adipocytes produce heat, counteracting hypothermia, obesity, and diabetes [1]. Brown fat utilizes high mitochondrial content and high mitochondrial uncoupling protein 1 (UCP-1) to uncouple respiration and dissipate chemical energy as heat [2–4]. Meanwhile, small amounts of brown adipose tissue (BAT) may be found in the neck; in supra-clavicular and axillary regions; in paravertebral, perirenal/adrenal, and paraventral regions; around major vessels (the aorta and its main branches: carotids, subclavian, intercostals, and renal arteries). BAT can also be found within white adipose tissue (WAT) and skeletal muscle tissues [5]. Notably, histological studies on humans suggest that brown and white adipocytes are mixed together [6–8]. Brown fat cells are characterized by multilocular lipid droplets and increased amount of mitochondria which express UCP-1 [9]. UCP-1 is located on the inner membrane of mitochondria and uncouples the rates of substrate oxidation and ATP production by favoring the loss of protons and subsequent energy release [10].

Cyclooxygenase (COX) catalyzes the rate-limiting step of prostanoid biosynthesis. Two COX isoforms have been identified, COX-1, the constitutive form, and COX-2, the inducible form [11]. COX-2 is implicated in body fat regulation, but underlying cellular mechanism remains to be elucidated [12].

In the present study, burn injury model was constructed to explore the influences of Celecoxib, an inhibitor of COX-2, on fat catabolism and hypermetabolism after burn, as well as relevant molecular mechanisms.

## Methods

### Burn injury model and grouping

The present study was approved by Subcommittee on Research Animal Care of the First Affiliated Hospital to PLA General Hospital. A total of 100 Balbe/c mice (male, 20 ± 3 g) were randomly divided into four groups: sham treated group (S), burned group (B), sham burn + Celecoxib group (C), and burn + Celecoxib group (BC), 25 in each group. The animal experiments were performed in the Animal Experimental Center of the First Affiliated Hospital to PLA General Hospital.

Thermal injury was produced in clean bench according to published protocols [13,14], with minor modifications. Each mice was anesthetized with pentobarbital sodium (50 mg/kg body wt, ip). After clipping back hair of the trunk, the animal was placed in a mold exposing 30% of total body surface area (TBSA), and the exposed area, which did not include the region expressing BAT, was immersed in 90°C water for 9 s, producing a full-thickness, third-degree thermal injury of 30% TBSA. Sham burn animals were similarly treated, with the exception that they were immersed in room temperature water. After burn or sham burn treatment, all animals immediately received fluid resuscitation with 40 ml/kg saline intraperitoneally. For mice in sham burn + Celecoxib group and burn + Celecoxib group, 1500 ppm Celecoxib normal saline was given through gavage [15,16]. All mice were caged individually throughout the study duration.

### Measurement of rectal temperatures

Rectal temperature of the mice in the four detected groups (S, B, C and BC) were detected. After the animal was placed on a hard surface, the probe was inserted, and rectal temperature was recorded after it was stabilized.

### Histology

On post-burn day 10, mice were killed by pentobarbital sodium anesthesia. BAT lobules and contiguous or nearby normal WAT tissues were excised from posterior cervical-upper thoracic region and immersed in 10% formalin. After 24 h of fixation, excised fat was examined, comparative changes were noted, and lobe sizes were measured. Tissues were then block sectioned, inserted into cassettes, processed into paraffin blocks, microtome-sectioned into 6 μm and stained with Hematoxylin and Eosin (H&E) for microscopic examination. HE slides were microscopically evaluated for histological changes in BAT and adjacent WAT tissues. Lipid content was estimated as a percentage of ‘clear areas’ relative to remaining areas in stained cellular components (nucleus and cytoplasm) and supporting connective tissue. A calibrated ocular grid was used for random fields, and percentages were calculated as statistical averages. This method was utilized in lieu of fat-stained frozen tissue sections cumbersome in evaluation and fraught with a host of staining artifacts. Additionally, this method guaranteed equal or higher accuracy.

### Transmission electron microscopy protocol

BAT was isolated from BALB/c mice of each group and washed twice with PBS solution. Then the tissues were fixed wtih 2% glutaraldehyde for 4 weeks. Later, the tissues were sectioned into 6 μm using microtome, and the ultrastructure, endoplasmic reticulum, and mitochondria of the cells were observed adopting transmission electron microscopy (TEM).

### Measurement of energy expenditure via indirect calorimetry

Indirect calorimetry (TSE systems) was performed for 24 h at 7th day after burn treatment. The animals were fasted overnight. No food was offered in metabolic chamber during measurements. The metabolic chamber was controlled by a computer system. The rates of volume of oxygen uptake (VO_2_) and volume of carbon dioxide output (VCO_2_) were recorded, and respiratory exchange ratio (RER) and energy expenditure (EE) were computed automatically. Resting values for each parameter were defined as the 10th percentile of raw data. The animals were given free access to water during the measurements.

### Isolation of mitochondria from iBAT

Mitochondria were isolated from fresh iBAT using mitochondria isolation kit (Sigma, St. Louis, MO). Briefly, tissue was washed with extraction buffer containing 50 mM HEPES, pH 7.5, 1 M mannitol, 350 mM sucrose, and 5 mM EGTA and cut into small pieces. The tissue was then homogenized in extraction buffer containing 5 mg/dl fatty acid-free BSA and centrifuged at 600 ***g*** for 5 min. Supernatant was centrifuged at 11000*** g***, and pellet was resuspended in extraction buffer and centrifuged at 60 ***g*** for 5 min. The supernatant was centrifuged at 11000 ***g***, and the pellet was suspended in a small volume of storage buffer containing 50 mM HEPES, pH 7.5, 1.25 M sucrose, 5 mM ATP, 0.4 mM ADP, 25 mM sodium succinate, 10 mM K_2_HPO_4_, and 5 mM DTT. All these procedures were performed in a cold room. The concentration of mitochondrial protein was determined through bicinchoninic acid method at 4°C. The samples were kept in a -80°C freezer until protein analysis.

### Measurement of BAT UCP-1 and COX-2 mRNA with quantitative real-time polymerase chain reaction

RNA was extracted from iBAT tissues using QUAZO L reagent (Gibco-BRL). Briefly, tissues were put into a screw-cap vial half-full of Zircona beads (Biospec). Tissue and beads were placed in BeadBeater for 2 min. Lysate was transferred into a 2-ml tube where RNA was purified using Qiagen RNeasy kit after chloroform treatment. Specific primers for mouse UCP-1, COX-2 and *β-actin* (as a housekeeping gene) were designed by Invitrogen (California, U.S.A.). The primer sequences were as follows: UCP-1 forward: 5′-AGGGTTTGTGGCTTCTTTTC-3′, reverse: 5′-TGGTTGGTTTTATTCGTGGT-3′; COX-2 forward: 5′- GTGCCTGGTCTGATGATGTATG-3′, reverse: 5′-TGAGTCTGCTGGTTTGGAATAG-3′; β-actin forward: 5′- AGAGGGAAATCGTGCGTGAC-3′, reverse: 5′-AGGAGCCAGGCAGTAATC-3′. Real time RT-PCR quantified UCP-1 and COX-2 mRNA using Cycle iQ Muticolor Real Time PCR Detection System (Bio-Rad, Hercules, CA) and SYBR Green PCR Master Mix (Applied Biosystems, Foster City, CA). To normalize variations in mRNA extraction and cDNA synthesis, the expression of *β-actin*, a housekeeping gene, was also measured. Thermal cycling conditions referred to an initial 94°C for 10 min, and followed by 50 cycles of 94°C for 30 s, 60°C for 30 s, and 72°C for 30 s. Relative expression of UCP-1 and COX-2 was normalized to β-actin, and calculated using the method of 2^−∆∆*C*_t_^.

### Western blotting for UCP-1 and COX-2 proteins’ expression

iBAT mitochondrial protein (20 g) was boiled in sample buffer (62.5 mM Tris/HCl, pH 6.8, 25% glycerol, 2% SDS, 0.01% Bromophenol Blue, 710 mM mercaptoethanol), separated by SDS/PAGE, and transferred on to nitrocellulose membranes. The membranes were blocked with LI-COR blocking buffer (diluted 1:1 in PBS; LI-COR Biosciences, Lincoln, NE) for 1 h, followed by overnight incubation with primary antibody. Primary antibodies were used at the following dilutions: anti-UCP-1 rabbit monoclonal antibody (Sigma–Aldrich, St. Louis MO) 1:1000, anti-COX-2 rabbit monoclonal antibody (Sigma–Aldrich, St. Louis MO) 1:1000, anti-glyceraldehyde-3-phosphate dehydrogenase (GAPDH: a housekeeping protein) rabbit monoclonal antibody (Sigma–Aldrich) 1:1000. After four washes with PBS-Tween 20 (PBS-T; 5 min each), the membranes were incubated with secondary antibody conjugated to horseradish peroxidase (HRP) in 1:1000 blocking buffer for 1 h at room temperature. After four washes with PBS-T (5 min each) and two washes with PBS (5 min each) at room temperature, immunoreactivity was visualized and quantified. Densitometry values for anti-UCP-1 and anti-COX-2 blots were normalized to anti-GAPDH controls.

### Statistical analysis

All results were presented as mean ± SEM. Differences in continuous variables between two groups were estimated via unpaired *t* test. Nonlinear regression analysis was employed to identify correlations between continuous data. Two-way ANOVA was employed to compare data among three or more groups, and individual means were adjusted through Bonferroni test. All statistics were performed using SPSS 17.0. Differences with *P* value less than 0.05 were considered to be significant.

## Results

### Morphological change in BAT induced by burn injury

**Figure 1:**
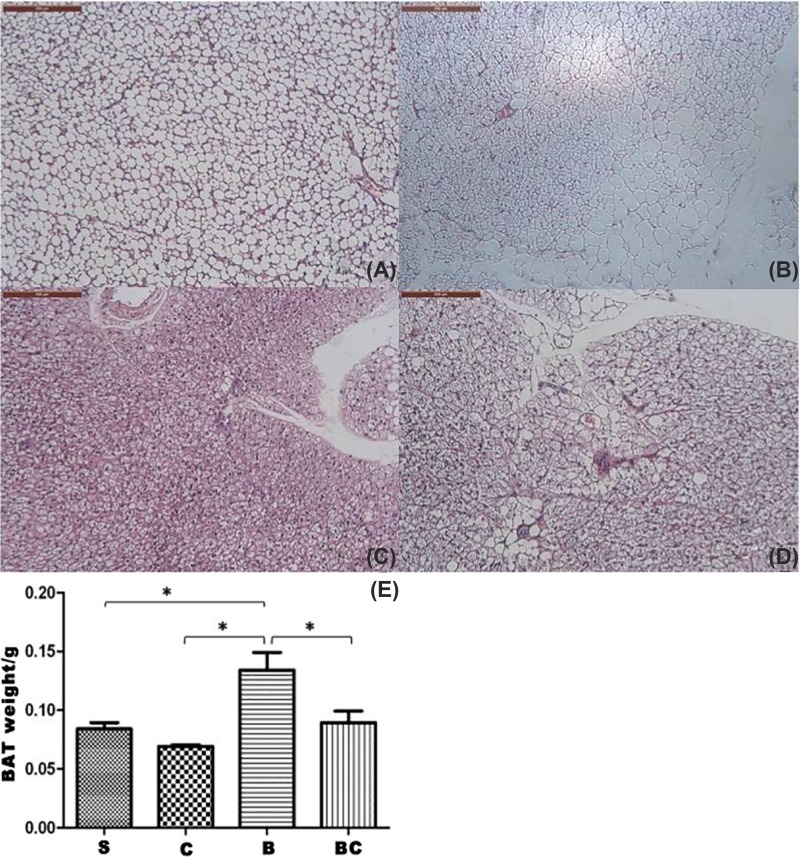
Burn injury-induced morphological changes in iBAT Gross views on interscapular fat pad from sham burn (**A**), sham Celecoxib (**B**), burn saline (**C**), and burn Celecoxib (**D**) animals. (**E**) Showed the weight of isolated BAT in interscapular area in mice. *: *P*<0.05.

We conducted tissue analyses to identify burn injury-associated iBAT activation. At macroscopic level, iBAT was much darker in burned animals than in sham-treated controls. However, in sham burn animals, it was difficult to distinguish iBAT from interscapular fat pad, which contains both iBAT and interscapular c (iWAT) based on gross observation. For further differentiation, we sectioned tissues, stained them with H&E, and performed histological analysis. At both low- and high-power light microscopic levels (Figure 1A–D), two populations of adipocytes, white and brown, coexisted in iBAT in sham burn animals. Multilobular fat vacuoles were prominent in brown adipocytes in sham burned animals (Figure 1A,C) and occupied the majority of cell volume. There was little Eosin-stained cytoplasm around fat droplets, and nucleus was located in peripheral area for each cell. In contrast, Eosin-stained cytoplasm was prominent in brown adipocytes from burned rats, and nucleus was centrally located and surrounded by cytoplasm (Figure 1B,D).

**Figure 2:**
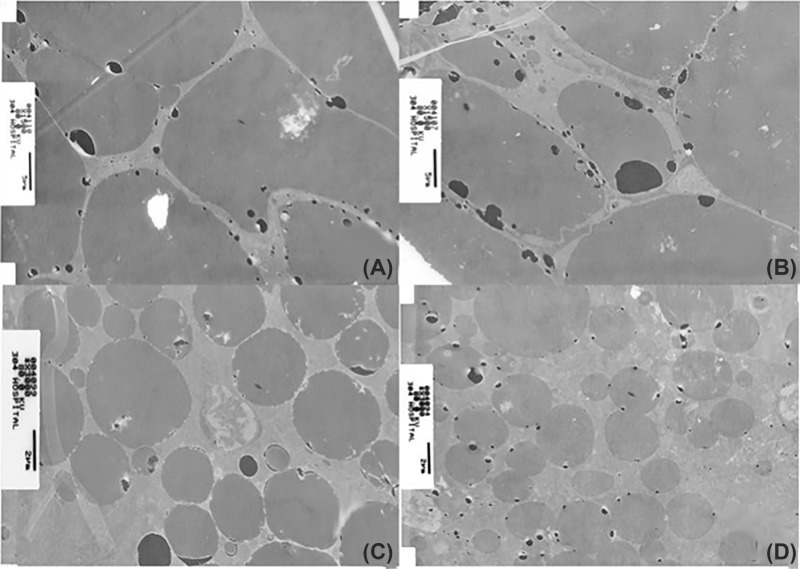
Ultrastructure of brown adipocytes in iBAT (**A**) (×1000) and (**C**) (×3000) from sham burn animals. (**B**) (×1000) and (**D**) (×3000) from burned animals.

The size of fat droplets in burned animal (Figure 1B,D) was much reduced compared with typical multilobular fat vacuoles seen in sham burn animals (Figure 1A,C). Isolated BAT in interscapular area was weighted for mice. As shown in Figure 1E, BAT weight was significantly heavier in burned group (*P*<0.05), and Celecoxib treatment could obviously reduce BAT weight (*P*<0.05). Therefore, burn injury was associated with iBAT activation and increased the density of brown adipocytes in interscapular area. The ultrastructure of brown adipocytes was also evaluated using TEM. In sham burn animals (Figure 2A), fat droplets occupied the majority of cytoplasm, and round-shaped small mitochondria were scattered in cytoplasm. In burned animals (Figure 2B), fat droplets were relatively small and cytoplasm was tightly packed by mitochondria. As illustrated in Figure 2C,D, morphometric analysis indicated that the ratio of fat droplet area to cytoplasmic area was significantly decreased after burn injury (68.41 vs. 14.41%, *P*<0.001). In addition, the number of mitochondria per brown adipocyte was increased after burn injury.

### Effect of celecoxib on treatment of burn-induced hypermetabolism

**Figure 3:**
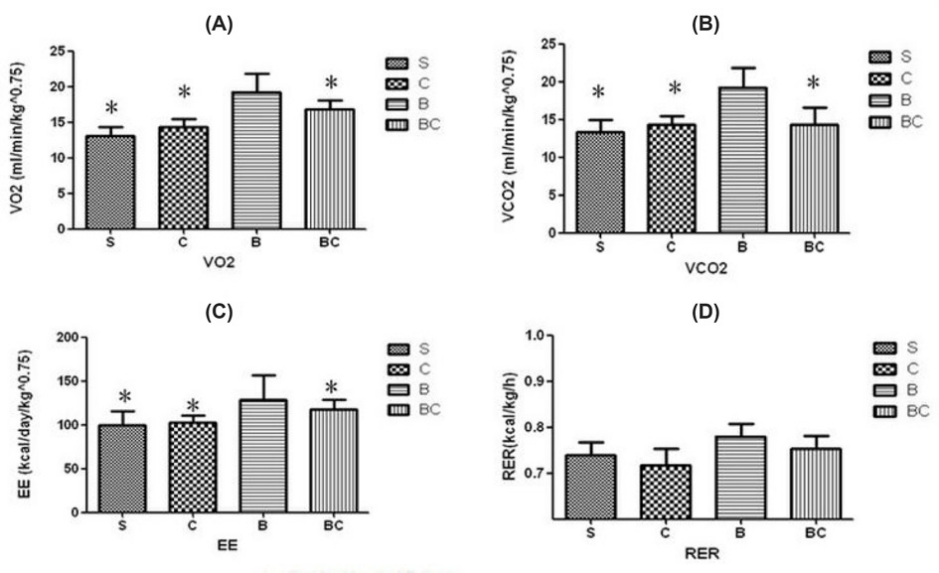
The effect of Celecoxib in reducing burn injury-induced hypermetabolism (**A**) VO_2_ was, increased in B group compared with S, C and BC groups. (**B**) VCO_2_ was, increased in B group compared with S, C and BC groups. (**C**) EE was, increased in B group compared with S, C and BC groups. (**D**) RER had no significant difference among four groups. B group: burned animals; S group: sham treated animals; C group: sham burn + Celecoxib animals; BC group: burn + Celecoxib animals; *P<0.01.

The effect of Celecoxib in reducing burn injury-induced hypermetabolism was further investigated in groups of sham burn and burned animals receiving continuous saline or Celecoxib infusion via implanted osmotic pumps. We explored metabolic rates of burned animals receiving 7 days of continuous Celecoxib infusion. The results are summarized in Figure 3. Two-way ANOVA demonstrated that in sham burn animals Celecoxib treatment did not cause significant difference in VO_2_, VCO_2_, or EE. The analysis also unveiled significantly increased metabolic rate after burn injury.

### Celecoxib treatment lowered the expressions of UCP-1 and COX-2

**Figure 4:**
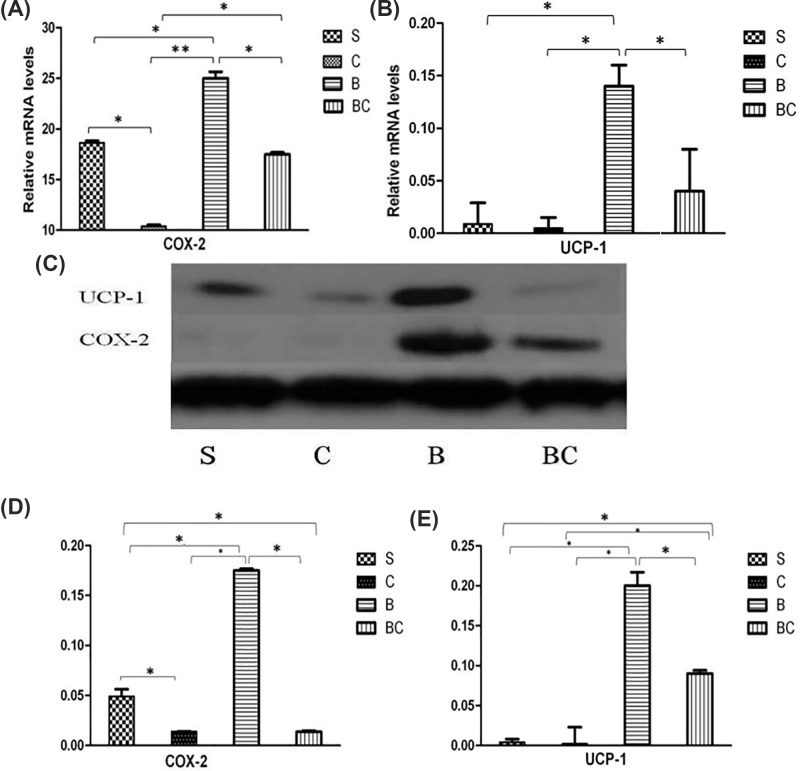
Expressions of UCP-1 and COX-2 mRNA and protein in iBAT Relative expressions of COX-2 (**A**) and UCP-1 (**B**) mRNA in BAT samples from the four groups. Representative Western blotting images for UCP-1 and COX-2 proteins in iBAT (**C**). Densitometry values were calculated based on GAPDH expression, and shown in (**D,E**). Values were shown as mean ± SEM in densitometry units. *P<0.05.

Possible mechanism of Celecoxib reducing EE in burned animals was investigated through its effects on UCP-1 and COX-2 expressions. Quantitative real-time polymerase chain reaction (qRT-PCR) analysis suggested that compared with S (sham burn) group, the expression of COX-2 was significantly increased in B (burn) group (*P*<0.05). The treatment with Celecoxib could obviously suppress COX-2 expression in both sham and burn groups (*P*<0.05 for both). Moreover, compared with S group, the level of COX-2 did not show obvious changes in BC (burn + Celecoxib) group (*P*>0.05), suggesting that Celecoxib treatment could inhibit COX-2 expression during burn (Figure 4A). qRT-PCR analysis on UCP-1 suggested that burn treatment could induce the expression of UCP-1, and that Celecoxib treatment was able to suppress UCP-1 expression (*P*<0.05 for both). Moreover, UCP-1 level did not show significant differences between S and BC groups (*P*>0.05), revealing that Celecoxib treatment might completely suppress UCP-1 activation induced by burn (Figure 4B).

In addition, the protein levels of UCP-1 and COX-2 in isolated mitochondria from iBAT were estimated using Western blot. Represented blotting images were shown in Figure 4C. Quantitative analysis demonstrated that compared with S group, burn treatment (B group) significantly induced the expression of COX-2 (*P*<0.05). Celecoxib could obviously inhibit COX-2 expression in both sham and burn groups (*P*<0.05 for both). Moreover, the protein levels of COX-2 in C and BC groups did not show significant difference (*P*>0.05), revealing that Celecoxib treatment inhibited COX-2 expression during burn (Figure 4D). As for UCP-1 protein in isolated mitochondria from iBAT, B group showed a significantly increasing tendency compared with S group (*P*<0.05). Celecoxib could obviously inhibit UCP-1 expression in both sham and burn groups (*P*<0.05 for both). Moreover, UCP-1 expression did not show obvious differences between C and BC groups (*P*>0.05), suggesting an inhibiting effect of Celecoxib treatment on UCP-1 expression during burn (Figure 4E).

## Discussion

Two major observations were completed in two studies in this investigation. First, BAT was activated by burn injury and was associated with increased expression of UCP-1 and augmented amount of mitochondria. Second, mitochondria-targeted peptide Celecoxib attenuated burn injury-induced hypermetabolism, which was correlated with decreased expression of COX-2 and UCP-1. These observations clearly demonstrated that burn injury significantly increased resting EE at 7th day after burn. It was worth mentioning that in study 2, animals underwent surgical procedures for the implantation of catheters and Celecoxib delivery pumps; however, both burned groups showed similar increments in EE compared with sham burn animals, indicating that the above-mentioned surgical procedures did not exacerbate hypermetabolism at 7th day following burn injury. Thus, the observed alterations in metabolic rate reflected hypermetabolism induced merely by burn injury.

Our previous study [17] revealed morphological changes in BAT after burn injury. The present study further explored those changes through immunohistochemistry and TEM, confirming that burn injury increased BAT mitochondria. All the observed changes were correlated with increased mitochondrion biogenesis and lipolysis. Increased mitochondrion biogenesis and lipolysis in BAT might be a possible mechanism for the development of burn injury-induced hypermetabolism.

In the present study, mechanisms for increased BAT energetics were further studied through measuring UCP-1 and COX-2 expressions in isolated mitochondria from BAT. In the present study, we observed a significant alleviation in hypermetabolism following Celecoxib treatment in burned animals. The present study also revealed that reduction in resting EE was correlated with the reduction in brown adipocytes and reduced UCP-1 expression. These findings provided further evidence supporting that BAT and UCP-1 were associated with burn injury-induced hypermetabolic state. Mechanism underlying the effect of Celecoxib on energy metabolism can be explained by its role in modulating mitochondrial function after thermal injury. Significant increase in superoxide level following burn injury and oxidative damage to tissues are implicated in inflammation, systemic inflammatory response syndrome, severe injury, infection, sepsis, and multiple organ failure. Recent studies have demonstrated that superoxide induces uncoupling process in mitochondria, and that uncoupling is correlated with UCP-1 expression in different tissues, but not in those not expressing UCPs, such as liver [18–20]. The expression of UCP-1 in BAT occurs in mitochondria and is a nucleotide-sensitive process [21]. Mitochondria-targeted antioxidants could abolish superoxide-induced uncoupling through lowering UCP-1 expression [22]. Celecoxib is a scavenger of ROS that ameliorates lipid peroxidation, reduces mitochondrial ROS levels, inhibits mitochondrial permeability transition, and prevents the swelling of isolated mitochondria [23,24]. In addition, COX-2 is an essential factor for UCP-1 synthesis, which is a necessary product to induce the transformation of white adipocytes into brown adipocytes. Celecoxib is an inhibitor of COX-2, and the treatment with Celecoxib could suppress the expressions of COX-2 and UCP-1, thus inhibiting BAT activation and hypermetabolism. Taken together, mechanism for Celecoxib functioning was associated with reducing burn injury-induced hypermetabolism which was mediated by the inhibition of superoxide-induced UCP-1 expression in BAT.

In recent years, BAT has become a target tissue in developing strategies for treating diseases associated with hypometabolic states such as diabetes and obesity [25]. The present study demonstrated that BAT might also play a role in burn injury-induced hypermetabolism. Therefore, BAT might be a potential target in treating burn injury-induced hypermetabolism. Ultrastructural analysis on BAT could serve as an indicator for treatment response in both hypo- and hypermetabolic diseases and injuries. In conclusion, our studies have demonstrated that burn injury-induced hypermetabolism is associated with the activation of BAT with significant up-regulation of UCP-1 expression and mitochondria biogenesis. The inhibition of this hypermetabolic state by Celecoxib may be related to reduced mitochondrial UCP-1 expression. Therefore, altered mitochondrial function and increased uncoupling process are possible important contributors to burn injury-induced hypermetabolism. In the future, alteration in BAT could be a therapeutic target in reducing hypermetabolism and associated protein wasting in metabolic care of severely burned patients.

Limitations in the present study should be noted. First, the sample size was not large enough. Second, the influences of Celecoxib treatment on the ultrastructure of brown adipocytes were not estimated by TEM in our study, which might reduce the veracity of final result. Third, mechanism for Celecoxib treatment functioning on burn-induced hypermetabolism was not explored. In addition, the influences of Celecoxib on COX-1, the constitutive form of COX, were not investigated in our study. Besides, Celecoxib could inhibit SUP-1 expression via multiple ways, but whether other inhibitors of COX-2 could suppress the expression of UCP-1 was not estimated. Therefore, further studies would be necessary in the future to verify our findings and investigate relevant mechanisms.

## Abbreviations

AT: adipose tissue
BAT: brown adipose tissue
COX-2: cyclooxygenase-2
EE: energy expenditure
GAPDH: glyceraldehyde-3-phosphate dehydrogenase
H&E: Hematoxylin and Eosin
PBS-T: PBS-Tween 20
qRT-PCR: quantitative real-time polymerase chain reaction
RER: respiratory exchange ratio
TBSA: total body surface area
TEM: transmission electron microscopy
UCP-1: uncoupled protein-1
VCO_2_: volume of carbon dioxide output
VO_2_: volume of oxygen uptake
WAT: white adipose tissue
